# Prevalence and Determinants of Current Cigarette Smoking Among Adolescents in Thailand: Evidence From 2021 Global School-Based Health Survey

**DOI:** 10.34172/jrhs.2024.145

**Published:** 2024-06-01

**Authors:** Yusuff Adebayo Adebisi, Theerapon Phungdee, Surasak Saokaew, Don Eliseo Lucero-Prisno

**Affiliations:** ^1^College of Social Sciences, University of Glasgow, Glasgow, UK; ^2^Research Department, Global Health Focus, Thailand; ^3^Faculty of Education, Kasetsart University, Bangkok, Thailand; ^4^Division of Social and Administrative Practice, Department of Pharmaceutical Care, School of Pharmaceutical Sciences, University of Phayao, Phayao, Thailand; ^5^Center of Health Outcomes Research and Therapeutic Safety (Cohorts), School of Pharmaceutical Sciences, University of Phayao, Phayao, Thailand; ^6^Department of Global Health and Development, London School of Hygiene and Tropical Medicine, United Kingdom; ^7^Office for Research, Innovation, and Extension Services, Southern Leyte State University, Sogod, Southern Leyte, Philippines; ^8^Center for University Research, University of Makati, Makati City, Philippines

**Keywords:** Tobacco smoking, Adolescents, Prevalence, Determinants, Thailand

## Abstract

**Background:** Adolescent cigarette smoking remains a concern globally, including in Thailand. This research aimed to elucidate the prevalence and determinants of cigarette smoking among Thai adolescents.

**Study Design:** A cross-sectional study.

**Methods:** A cross-sectional analysis of data from the 2021 Thailand Global School-Based Health Survey with 5545 adolescents aged 13–17 with complete information was conducted on their cigarette smoking status. Bivariate analysis and multivariable logistic regression were performed to discern the determinants of tobacco smoking among adolescents.

**Results:** The overall weighted prevalence of cigarette smoking was 11.5% (95% confidence interval [CI]=9.7%, 13.5%), with adolescent males at 18.2% (95% CI=15.3%, 21.4%) and adolescent females at 5.6% (95% CI=4.2%, 7.4%). The multivariable logistic regression also revealed that males were more likely to be smokers (Adjusted Odd Ratio [AOR]=1.58; 95% CI=1.02, 2.45, *P*=0.040) compared to females. The presence of smokers in their vicinity significantly increased the odds of smoking (AOR=2.21, 95% CI=1.46, 3.36, *P*<0.001). Current alcohol use (AOR=3.37, 95% CI=2.21–5.14, *P*<0.001) and current marijuana use (AOR=4.53, 95% CI=2.06, 9.99, *P*<0.001) were both significant determinants of smoking. Notably, early initiation of cigarette use (before age 14) was associated with a lower likelihood of current smoking (AOR=0.54, 95% CI=0.33, 0.92, *P*=0.022).

**Conclusion:** With an overall prevalence of smoking among adolescents at 11.5%, our study highlights a significant public health concern. The positive determinants of the identified tobacco smoking include being male, having smokers in their vicinity, and currently using alcohol, and marijuana, while early initiation of cigarette use before age 14 is identified as an inverse determinant.

## Background

 Cigarette smoking stands as a leading cause of preventable diseases worldwide, responsible for approximately 8 million deaths annually.^1^ Globally, an estimated 1.3 billion people indulge in tobacco use, with a disproportionate number of smoking-related deaths occurring in low- and middle-income countries.^2,3^ This global health crisis suggests the urgency of addressing the smoking epidemic, not only as a matter of public health but also as a critical socio-economic concern, given its vast impact on healthcare systems and global productivity.^1-3^

 In Thailand, the smoking issue is particularly pressing.^4,5,6^ Over 19% of the population aged 15 years and older are smokers, with a concerning trend observed among adolescents.^7^ Approximately 7.8% of Thai adolescents aged 15–18 years are regular smokers, and alarmingly, about 20% of these young smokers began their smoking habits before the age of 10.^8^ This early initiation into smoking poses significant challenges for public health interventions and implies the need for early and effective preventative strategies. The susceptibility of youth to smoking is a global concern, with variations across regions.^9^ In the World Health Organization (WHO) South-East Asia region, for instance, 10.1% of never-smoking youth are susceptible to smoking, a percentage that soars to 29.8% in Europe.^9^ This susceptibility is influenced by a myriad of factors, including peer pressure, exposure to tobacco advertising, and socio-environmental determinants.^10^ Understanding these factors is important in developing targeted interventions to prevent smoking initiation among adolescents.

 The health and economic implications of smoking are profound in Thailand.^11^ Diseases such as chronic obstructive pulmonary disease, lung cancer, and cardiovascular disease, often attributed to smoking, significantly contribute to the country’s health burden.^11^ Economically, the cost of smoking in Thailand is substantial, accounting for an estimated 0.5% of the gross domestic product.^12^ This financial burden includes direct healthcare costs and indirect costs related to lost productivity due to smoking-related morbidity and mortality.^13^ Given these significant health and economic impacts, identifying the determinants of adolescent smoking in Thailand has become particularly critical. By understanding factors such as gender, exposure to smoking, and substance use that influence smoking behaviors in adolescents, targeted interventions can be developed to curb the initiation and prevalence of smoking in this vulnerable group. Early prevention and intervention strategies aimed at these determinants are essential not only for reducing the immediate health risks to adolescents but also for mitigating the long-term economic and health burdens on Thai society. Such strategies could include educational programs in schools, stricter enforcement of underage smoking laws, and public health campaigns aimed at reducing the social acceptance of smoking among adolescents.

 This research aims to elucidate the prevalence and determinants of cigarette smoking among Thai adolescents, leveraging data from the 2021 Global School-Based Health Survey. By analyzing this demographic, the study seeks to identify key factors contributing to smoking initiation and persistence among Thai youth. The findings are anticipated to provide valuable insights for policymakers and public health officials in designing effective tobacco control strategies and interventions, specifically tailored to address adolescent smoking behaviors in Thailand. This study not only contributes to the existing body of knowledge on tobacco use but also serves as an important step toward mitigating the public health crisis posed by smoking among youth.

## Method

###  Study design, data source, and participants

 This study involves a secondary analysis of the 2021 Global School-based Student Health Survey (GSHS) data, representative of students in grades 7–12 across Thailand.^14^ The 2021 survey marks the third execution of the GSHS by Thailand. The original data were collected using a two-stage cluster sample design, beginning with the selection of schools based on enrolment size and followed by random class selection within these schools. The stratified two-stage cluster sampling method is particularly well-suited for surveys such as the GSHS because it allows for efficient data collection across diverse populations while ensuring that the sample is representative of the broader student population. The method’s design takes into account the balance between statistical power and practical feasibility, considering factors such as anticipated response rates, desired precision levels for estimates, and the heterogeneity of the student population. Targeting adolescents aged 13–17 years, the survey achieved a school response rate of 92% and a student response rate of 90%, resulting in an overall response rate of 83%. Detailed information on the study methodology, sampling, and procedures has been detailed elsewhere.^14^ School administrators had the option to not involve their schools in the study, and students could choose whether to fill out the questionnaire. Students were assured that their participation decision would not impact their academic grades or marks, and their responses would remain confidential. Additionally, to safeguard privacy, the names and locations of the schools involved are not disclosed in the public dataset.

 The GSHS employs self-administered questionnaires to capture a wide range of health behaviors and protective factors among young people, which is pivotal in understanding adolescent health globally. The questionnaire covered various domains such as alcohol and drug use, dietary behaviors, mental health, physical activity, sexual behaviors, tobacco use, and violence. Students meeting the inclusion criteria (aged 13–17 years, grades 7–12, and willingness to participate) and providing written informed consent were given questionnaires to complete. These questionnaires were administered on computer-scannable forms and distributed by trained staff during a class period. Data processing tasks, including scanning, cleaning, editing, and weighting, were executed at the WHO and the US Centers for Disease Control and Prevention (CDC). The Ministry of Public Health in Thailand spearheaded the survey with support from the WHO and CDC. In the current analysis, this rich dataset was utilized to examine specific aspects of adolescent health behavior, leveraging the extensive data collected to derive new insights and understandings. In writing this manuscript, we adhered to the guidelines outlined in the Strengthening the Reporting of Observational Studies in Epidemiology statement.^15^

###  Study variables

 A total of 5661 adolescents participated in the study. Smoking status was determined by a single survey question: “During the past 30 days, how many days did you smoke cigarettes?” Participants reporting ‘0’ days were classified as non-smokers, while those reporting one or more days were grouped as smokers. To ensure a complete case analysis, participants with missing responses for smoking status (n = 116) were excluded, resulting in 5545 adolescents available for subsequent analysis. For other covariates, where some missing values were present, these cases were also excluded from the respective analyses to maintain methodological consistency by using only complete cases.

 Other covariates, selected based on a theoretical understanding of predictors of tobacco smoking, were methodically coded to align with the nature of each variable. Demographic factors were categorized into two age groups (those under 15 years and those 15 years or older). Gender was categorized as male or female, and the educational level was divided into two brackets (grades 7–9 and 10–12). Social influences were coded binary (yes or no) and included factors such as exposure to smoking, parental or guardian tobacco use, lack of close friends, experiences of bullying in and outside of school, and unpermitted absences from classes or school. Similarly, psychological and emotional factors were coded as ‘Yes’ or ‘No’, asking participants if they had felt lonely or had trouble sleeping due to worry. Substance use variables were also binary, covering current use of alcohol and marijuana, past use of amphetamines or methamphetamines, and early initiation of substance use with questions about trying cigarettes or alcohol before the age of 14. This structured coding system allowed for the quantification of a diverse range of factors, from basic demographic details to more subjective psychological experiences, providing a comprehensive view of the variables that might influence smoking behavior among the participants.

###  Statistical analyses

 Descriptive statistics were utilized to summarize categorical variables, presenting them as frequencies and percentages. The prevalence of tobacco smoking was calculated by dividing the number of smokers by the total surveyed participants, then multiplying by 100 for a percentage. Weighted prevalence and 95% confidence intervals (CIs) were estimated and reported to accurately reflect the study’s design and adjust for potential non-response bias. Bivariate analysis, employing the chi-square test of independence, was also conducted to assess the associations between various covariates and smoking status (smokers and non-smokers).

 In our analytical approach, the criteria for variable inclusion in the multivariable logistic regression model were defined to ensure a robust examination of the determinants of cigarette smoking among adolescents. Variables were selected based on two primary criteria, namely, statistical significance in bivariate analyses and their theoretical relevance to adolescent smoking behaviors as identified in the existing literature. Specifically, variables that demonstrated a *P* value of less than 0.05 in bivariate analysis were considered candidates for inclusion in the multivariable model to identify potential predictors of smoking behavior. This statistical threshold was chosen to balance the inclusion of relevant variables while minimizing the risk of type I errors. Educational grade was not significant in the bivariate analysis and therefore was not included in the multivariable model. However, other variables were included in the multivariable model and were mutually adjusted for one another. The results from this regression were reported in terms of adjusted odds ratios (AORs), CIs, and *P* values. These metrics were also weighted to further adjust for non-response biasusing the*svy command*, thereby enhancing the representativeness of the findings. In the multivariable logistic model, covariates with missing values were included in the analysis without imputation, allowing the logistic regression procedure to automatically handle these cases through listwise deletion.

**Figure 1:**
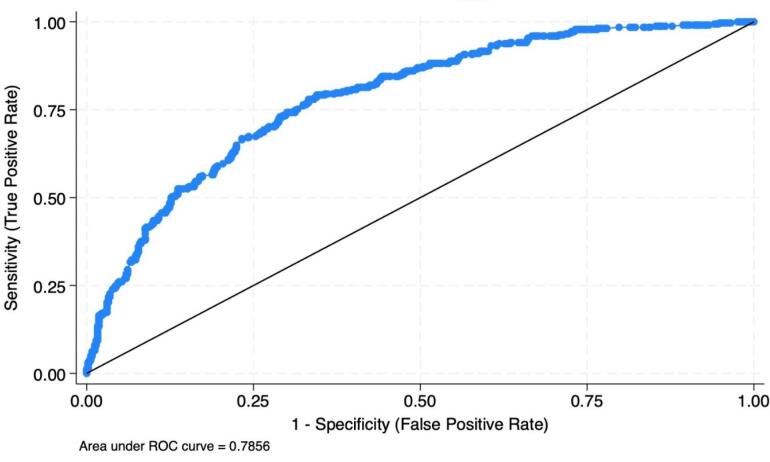
ROC Curve for Predicting Adolescent Smoking Behavior
*Note*. ROC curve: Receiver operating characteristic curve

 The evaluation of the final model included various aspects to confirm its fit and predictive accuracy. The Hosmer-Lemeshow test, with a chi-square statistic of 5.67 and a *P*-value of 0.6837, was used to assess the goodness-of-fit. The non-significant result suggests that the model fits the data well. Moreover, the model’s ability to discriminate between adolescents who smoke and those who do not was evaluated using the area under the receiver operating characteristic curve (Figure 1). An area under the curve value of 0.7856 was obtained, indicating that the model has a good discriminative capacity, with values closer to 1.0 reflecting a stronger ability to differentiate between the two groups. This combination of goodness-of-fit and discriminative ability underlines the model’s robustness in identifying key determinants of smoking behavior among Thai adolescents. Regarding multicollinearity among the covariates, it was observed that the variance inflation factor (VIF) values were well below the commonly used threshold of 5 or 10 [minimum VIF = 1.11, maximum VIF = 3.35, and mean VIF = 2.04], representing no significant multicollinearity issues that could affect the reliability of the regression model. A *P* value threshold of 0.05 was set for determining statistical significance, in line with standard practices in statistical analysis. All analyses were conducted using STATA, version 18.

 In the receiver operating characteristic curve, the X-axis represents the false-positive rate, which is the proportion of non-smoking adolescents incorrectly identified as smokers by the model. The Y-axis denotes the sensitivity or true-positive rate, indicating the correct identification of the proportion of actual smokers. The curve demonstrates the trade-off between correctly identifying smokers and incorrectly labeling non-smokers as smokers at various threshold settings.

## Results

 The weighted prevalence of cigarette smoking in the surveyed population was found to be 11.5% (95% CI = 9.7%, 13.5%), with 642 out of 5,545 participants identified as smokers. Additionally, the weighted prevalence of smoking among adolescent males was 18.2% (95% CI = 15.3%, 21.4%), while the weighted prevalence of smoking among adolescent females was 5.6% (95% CI = 4.2%, 7.4%).

Table 1 presents a breakdown of demographic, social, psychological, and substance use characteristics across smoker (n = 642) and non-smoker (n = 4903) groups, with a total sample size of 5,545 participants. Significant differences were observed in age distribution (*P*= 0.017), with smokers less likely to be under 15 years (42.2% vs. 47.2%). Gender distribution varied significantly (*P*< 0.001), with a higher proportion of males among smokers (71.3%) compared to non-smokers (40.6%). Factors such as smoking in the presence, parental/guardians’ tobacco use, lack of close friends, bullying at school, bullying outside school, and missing classes/school without permission all showed highly significant differences (*P*< 0.001) between smokers and non-smokers. In each of these categories, a higher proportion of smokers exhibited these behaviors or characteristics compared to non-smokers. Feelings of loneliness and worry leading to sleeplessness were significantly more common in smokers (*P*< 0.001). Smokers reported substantially higher rates of current alcohol use (62.1% vs. 19.7%, *P* < 0.001), marijuana use (27.4% vs. 1.4%, *P*< 0.001), amphetamine/methamphetamine use (18.7% vs. 1.3%, *P*< 0.001), and early initiation of cigarette and alcohol use (*P* < 0.001 for both) compared to non-smokers.

**Table 1:** Relevant characteristics by cigarette smoking status

**Characteristics**	**Smokers, n=642**		**Non-smokers, n=4903**		***P*** ** value**
**Demographic factor**	**Number**	**Percent**	**Number**	**Percent**	
Age (y)					0.017
< 15	271	42.2	2,314	47.2	
≥ 15	371	57.8	2,589	52.8	
Gender					0.001
Male	452	71.3	1,985	40.6	
Female	182	28.7	2,904	59.4	
Educational level					0.545
7-9	439	68.4	3,410	69.6	
10-12	203	31.6	1,493	30.4	
Social influence					
Smoking in their presence (Yes)	473	75.4	1,994	40.8	0.001
Parental/guardians tobacco use (Yes)	324	51.9	1,938	39.7	0.001
Did not have close friends (Yes)	61	9.8	291	6.0	0.001
Bullied at school (Yes)	169	29.3	975	20.1	0.001
Bullied outside school (Yes)	134	23.2	441	9.1	0.001
Missed classes or school without permission (Yes)	283	45.6	825	17.1	0.001
Psychological and emotional factor					
Felt lonely (Yes)	147	23.4	861	17.7	0.001
Worried and unable to sleep (Yes)	153	24.6	709	14.6	0.001
Substance use					
Current alcohol use (Yes)	371	62.1	951	19.7	0.001
Current marijuana use (Yes)	159	27.4	69	1.4	0.001
Ever amphetamine or methamphetamine use (Yes)	111	18.7	63	1.3	0.001
Tried cigarettes before age 14 (Yes)	440	69.6	671	77.9	0.001
Tried alcohol before age 14 (Yes)	293	65.1	1,015	51.8	0.001

Table 2 details the findings from a multivariable logistic regression analysis investigating the determinants of cigarette smoking among adolescents in Thailand. The analysis adjusted for various demographic, social, psychological, and substance use factors. Age was not a significant determinant (*P* = 0.450), with adolescents aged 15 or greater having an AOR of 0.83 (95% CI = 0.50–1.36) compared to those less than 15 years. However, gender was a significant determinant (*P*= 0.040), with males having an AOR of 1.58 (95% CI = 1.02–2.45) in comparison to females. The presence of smoking in their vicinity significantly increased the odds of smoking (AOR = 2.21, 95% CI = 1.46–3.36, *P* < 0.001). Other factors, such as parental/guardian tobacco use, lack of close friends, bullying at school, bullying outside school, and missing classes/school without permission, were not statistically significant. Neither feelings of loneliness nor worries leading to sleeplessness were significant determinants. Current alcohol use (AOR = 3.37, 95% CI = 2.21–5.14, *P* < 0.001) and current marijuana use (AOR = 4.53, 95% CI = 2.06–9.99, *P* < 0.001) were both significant determinants of smoking. Early initiation of cigarette use (before age 14) also emerged as a significant factor (AOR = 0.54, 95% CI = 0.33–0.92, *P* = 0.022). Other substance use factors, such as amphetamine or methamphetamine use and early alcohol initiation, were not significant.

**Table 2:** Determinants of cigarette smoking among adolescents in Thailand using multivariable logistic regression

**Determinant**	**Adjusted OR (95% CI)**	***P*** ** value**
Demographic factors		
Age (≥ 15/ < 15 years)	0.83 (0.50, 1.36)	0.450
Gender (Male/female)	1.58 (1.02, 2.45)	0.040
Social influence		
Smoking in their presence (Yes/No)	2.21 (1.46, 3.36)	0.001
Parental/guardians tobacco use (Yes/No)	0.75 (0.50, 1.11)	0.150
Did not have close friends (Yes/No)	1.17 (0.50, 2.72)	0.715
Bullied at school (Yes/No)	1.48 (0.89, 2.48)	0.132
Bullied outside school (Yes/No)	1.64 (0.92, 2.94)	0.095
Missed classes or school without permission (Yes/No)	1.34 (0.87, 2.08)	0.187
Psychological and emotional factor		
Felt lonely (Yes/No)	1.01 (0.59, 1.70)	0.187
Worried and unable to sleep (Yes/No)	0.77 (0.42, 1.41)	0.391
Substance use		
Current alcohol use (Yes/No)	3.37 (2.21, 5.14)	0.001
Current marijuana use (Yes/No)	4.53 (2.06, 9.99)	0.001
Ever amphetamine or methamphetamine use (Yes/No)	1.43 (0.66, 3.13)	0.367
Tried cigarettes before age 14 (Yes/No)	0.54 (0.33, 0.92)	0.022
Tried alcohol before age 14 (Yes/No)	0.80 (0.50, 1.29)	0.363

*Note*. OR: Odds ratio; CI: Confidence interval. Variables are mutually adjusted in the multivariable model.

## Discussion

 Adolescence is a stage marked by a natural inclination toward exploring new experiences and sometimes engaging in risky activities, including cigarette smoking.^16^ While not every adolescent who experiments with cigarettes continues to smoke regularly, initial experimentation often serves as a precursor to consistent tobacco use in the future.^16^ Interestingly, our findings also revealed that adolescents who initiated cigarette use early (before age 14) were less likely to be current smokers, suggesting that early experimentation does not always lead to continued smoking. This finding demonstrates the complexity of smoking behaviors and the potential for early interventions to encourage cessation before habits become entrenched.

 The prevalence of cigarette smoking among adolescents in our study revealed notable demographic determinants. Compared to past studies in Thailand,^4,17,18^ our findings align with the trend that smoking prevalence is higher in males than females. This gender disparity in smoking habits is not unique to Thailand; international studies,^19-21^ such as those conducted in the United States,^22^ Africa,^23^ and Europe,^24^ have also consistently shown higher smoking rates among adolescent males. Similar findings have been reported in a study that included 530 234 adolescents from 143 countries that had conducted at least one survey between 2010 and 2018, where the global prevalence of cigarette smoking was 11.3% in boys and 6.1% in girls, based on cigarette smoking on at least one day during the past 30 days.^25^ The overall prevalence of smoking (11.5%) in our study was higher than the previous estimates from national representative surveys conducted in Thailand between 1996 and 2015.^26^ The lack of a significant age effect in our study contrasts with some global trends where older adolescents tend to exhibit higher smoking rates.^27^ This discrepancy may reflect cultural or societal differences in Thailand, suggesting that interventions targeting young adolescents could be crucial in early prevention.

 Social influence played a substantial role in smoking behavior, with adolescents more likely to smoke if exposed to smoking in their presence. This finding is consistent with those of Thai studies^10,28^ and mirrors global patterns,^29-31^ where peer influence is a critical factor in adolescent smoking. However, unlike some international studies,^32-34^ parental smoking did not emerge as a significant factor in our study. This variance could be due to differing familial norms or tobacco control policies in Thailand. The lack of a significant impact from bullying and social isolation indicates these may not be primary drivers of smoking in Thai adolescents, which contradicts the findings of some Western studies, where these factors were more pronounced.^35,36^ Understanding these dynamics is key to developing targeted anti-smoking campaigns and interventions in Thailand. Tailored strategies that focus on peer group influence might be more effective in reducing smoking rates among Thai adolescents. Furthermore, incorporating educational programs that address the social aspects of smoking, including the desire to fit in with peers, could help mitigate the influence of peer pressure. In the Thai context, our findings also suggest that interventions should prioritize enhancing self-esteem and resilience among adolescents to combat the initiation of smoking.

 Surprisingly, psychological and emotional factors such as loneliness and sleep-related worries did not significantly influence smoking among Thai adolescents. This contrasts with the results of studies from other countries, where mental health issues often correlate with higher smoking rates.^37,38^ The divergence may be attributable to different coping mechanisms or societal attitudes toward mental health in Thailand. This finding suggests that while addressing mental health is crucial, it might not directly translate into reduced smoking rates among Thai adolescents, emphasizing the need for targeted smoking cessation strategies. Therefore, it becomes imperative to explore and understand the specific cultural and social contexts that drive smoking behaviors in Thai adolescents. Identifying these unique factors can help tailor prevention and cessation programs that resonate with their experiences and values. For instance, focusing on strengthening social bonds and community engagement could serve as effective deterrents against smoking initiation. Additionally, this insight underlines the importance of a multifaceted approach in public health strategies, where mental health support is part of a broader initiative rather than the sole focus in combating adolescent smoking.

 The strong association between smoking and other substance use, notably alcohol and marijuana, is a critical finding. This conforms to international literature,^39-41^ where substance use is often a comorbid behavior with smoking. Our study adds to the growing body of evidence suggesting a ‘gateway’ effect, where the use of one substance increases the likelihood of using others. This pattern is particularly concerning in the Thai context, given the relatively high prevalence of these behaviors. Effective prevention programs in Thailand must therefore address the broader context of substance use rather than focusing solely on cigarette smoking.

 Integrating comprehensive education about the risks associated with polysubstance use into school curricula and community programs could be a key strategy. By highlighting the interconnected nature of substance use behaviors, these programs can aim to reduce the initiation and co-use of alcohol, marijuana, and tobacco among adolescents. Moreover, adopting a holistic approach that includes family and community involvement can enhance the effectiveness of these interventions, creating a supportive environment that encourages healthier lifestyle choices. Additionally, collaboration with digital media platforms to spread awareness and counteract the glamorization of substance use can further reinforce the message among Thai youth.

 The study’s findings have significant implications for public health policy and adolescent health interventions in Thailand. The identified determinants of smoking highlight the need for gender-specific strategies and interventions that address the social environment of adolescents, especially in settings where smoking is prevalent. The apparent disconnect between psychological factors and smoking in our Thai cohort suggests that universal anti-smoking strategies may need cultural adaptation to be effective in different settings. Future research should explore the causal pathways between these determinants and smoking behavior, particularly focusing on longitudinal studies to understand how these relationships evolve over time. Recognizing the cultural aspect of smoking behavior among Thai adolescents is vital for crafting interventions that resonate with their specific experiences and social contexts. Tailoring anti-smoking campaigns to reflect the identified gender differences and social dynamics can enhance their relevance and impact. Additionally, the incorporation of community-based initiatives that engage peers, families, and schools in prevention efforts could foster a more supportive environment for adolescents to resist smoking. The emphasis on longitudinal research underscores the importance of monitoring changes and trends in smoking behavior over time, allowing for the dynamic adjustment of policies and programs to address emerging challenges effectively.

 This cross-sectional study offers a snapshot of the determinants of cigarette smoking among Thai adolescents, allowing for the analysis of a wide array of variables simultaneously. Its strength lies in its efficiency and ability to provide a representative overview of the current state of adolescent smoking in Thailand, which is crucial for informing public health strategies and policy decisions. However, the study’s design inherently limits the ability to establish causality between the identified factors and smoking behavior. Additionally, reliance on self-reported data may introduce recall bias, and the single point-in-time approach does not account for changes in behaviors or attitudes over time. Despite these limitations, the study provides valuable insights into the current landscape of adolescent smoking in Thailand, laying the groundwork for future longitudinal research to explore these associations further.

**Highlights**

- The study uncovered a worrying prevalence of cigarette smoking among Thai adolescents (11.5% overall weighted prevalence).
- Exposure to smoking in adolescents’ immediate environment and current substance use (alcohol and marijuana) emerged as significant determinants of smoking.
- Early initiation of cigarette use (before age 14) was inversely associated with current smoking.

## Conclusion

 In this study, a relatively high prevalence of smoking was found among adolescents, indicating a significant public health concern. The key determinants of tobacco smoking in this demographic included gender, with males more likely to smoke, and the influence of social environments, particularly the presence of smokers in their vicinity. Substance use patterns emerged as a critical factor; adolescents engaged in current alcohol and marijuana use were more inclined toward smoking. Early initiation of cigarette use, specifically before the age of 14, was another significant inverse determinant. These findings underscore the need for targeted intervention strategies focusing on these key determinants to effectively curb the rising trend of tobacco use among Thai adolescents. Moreover, our research highlights the urgency for comprehensive public health policies tailored to address the multifaceted nature of adolescent smoking behavior.

## Acknowledgements

 We thank all the students who participated in this study and the WHO for providing access to the data via https://extranet.who.int/ncdsmicrodata/index.php/catalog/946.

## Authors’ Contribution

**Conceptualization:** Yusuff Adebayo Adebisi.

**Data curation:** Yusuff Adebayo Adebisi.

**Formal analysis:** Yusuff Adebayo Adebisi.

**Investigation:** Yusuff Adebayo Adebisi, Theerapon Phungdee, Surasak Saokaew.

**Methodology:** Yusuff Adebayo Adebisi, Don Eliseo Lucero-Prisno.

**Project administration:** Yusuff Adebayo Adebisi, Surasak Saokaew, Theerapon Phungdee.

**Resources:** Yusuff Adebayo Adebisi, Surasak Saokaew, Theerapon Phungdee, Don Eliseo Lucero-Prisno.

**Software:** Yusuff Adebayo Adebisi.

**Supervision:** Don Eliseo Lucero-Prisno.

**Validation:** Yusuff Adebayo Adebisi, Don Eliseo Lucero-Prisno.

**Visualization:** Yusuff Adebayo Adebisi.

**Writing–original draft:** Yusuff Adebayo Adebisi.

**Writing–review & editing:** Yusuff Adebayo Adebisi, Surasak Saokaew, Theerapon Phungdee, Don Eliseo Lucero-Prisno.

## Competing Interests

 We declare no competing interests.

## Ethical Approval

 Although no separate ethical approval was required for this secondary analysis, the original survey was conducted with permission from the Ministry of Health, ensuring full compliance with ethical standards, including the acquisition of informed consent from school heads as well as parental consent and child assent for minors.

## Funding

 There was no source of funding for this research.
